# On doctor-patient relationship and feedback interventions

**DOI:** 10.1007/s40037-012-0030-3

**Published:** 2012-11-01

**Authors:** Onno T. Terpstra

**Affiliations:** Leiden University Medical Center, Leiden, the Netherlands

The first consultation with a patient is the beginning of a doctor-patient relationship. It is thus of major importance to conduct this in a correct and proper way. Consulting with a patient is a complicated skill that is gradually learned during medical training and perfected when one grows into one’s role as a doctor. The importance of an adequate consultation constitutes a strong argument to start learning this skill early in medical training. Hegge et al. [1] describe the positive results of a longitudinal training project to teach this skill during clerkships. Almost three-quarters of the students were satisfied with the educational approach (practising with simulated patients) and felt well prepared to conduct their consultations with real patients. However, the authors rather easily brushed aside the disadvantages of the simulated patient situation felt by others, referring to studies that compared simulated patients with real patients and ‘mostly found no difference’. Using simulated patients indeed has advantages for instructiveness and for standardizing possibilities for testing performance. But what about the emotional impact, which is an intrinsic part of learning to become a doctor? Students may prefer ‘the real thing’.

To distinguish the effects of communication between specialists and patients with medically unexplained physical symptoms (MUPS) on patient outcomes and use of health care, Weiland et al. [2] from four Dutch university medical centres performed a literature overview. Research in this field is limited and the articles included discuss different types of MUPS patients and describe different elements of communication strategies used by medical specialists. Nevertheless, their synthesis of data demonstrates that positive doctor-patient interaction and positive feedback from the doctor improves long-term coping with complaints as well as reducing use of health care.

Schönrock et al. [3] suggest improvements of the (Cleveland) clinical teaching effectiveness instrument (CTEI) arguing that student perceptions of teaching quality are vital for optimizing teaching quality, which in turn may result in better learning outcomes and thus in an improvement of patient care. To assess the quality of the CTEI the authors advocate the use of separate scales for frequency of teaching and quality of teaching. They note an intermingling in these rating scales that they suspect to affect their outcomes, which may be an obstacle in providing concrete and accurate feedback to faculty.

Three medical universities in the Netherlands wish to develop a shared question database to assess clinical reasoning of undergraduate students using Computer Based Assessment. To answer the question as to what might be the preferred question types to do so, Van Bruggen et al. [4] conducted a literature study. They consider a combination of Comprehensive Integrative Puzzle and Extended Matching Question most suitable.

The mantra that feedback intervention always improves performance is disputable. In this issue of PME, Olde Bekkink et al. [5] report their study on feedback as a didactical tool to enhance the effect of an interim assessment in a pathology course for second-year medical students. In their previous study an interim assessment improved the score in the examination finalizing the course [6]. Based on literature research, in their follow-up study the authors wanted to determine (i) whether explicit feedback *following* an interim assessment had an effect on the formal examination course and (ii) whether the effect of feedback was influenced by gender. Their data did not support either of these hypotheses. Reading between the lines the authors seem a little surprised when concluding that the feedback intervention did not result in better marks at the final examination. Subsequently they embark on a fairly extensive discussion of the literature on feedback focussing on the complexity of feedback and the many different factors that are involved.

Olde Bekkink et al. refer to a systematic review by Hattie and Timperley [7], who proposed a model of feedback that was meant to enhance learning at different levels (task, process, self-regulation, *the* self). But they do not mention that Hattie and Timperley extensively discussed the meaning of feedback. It is conceptualized as ‘information provided by an agent (e.g. teacher, peer, book, parent, self, experience) regarding aspects of one’s performance’. A teacher or parent can provide corrective information and encouragement, a peer can provide an alternative strategy, a book can provide information to clarify ideas, and a learner can look up answers to evaluate the correctness of responses. Hattie and Timperley highlighted that effect sizes reported in overviews demonstrate a considerable variability in the effect of feedback interventions. They referred to the extensive meta-analysis by Kluger and DeNisi [8]. These authors suggested that feedback intervention improved performance on average but that over one-third of the feedback interventions *decreased* performance. Furthermore, results suggest that the effectiveness of feedback intervention decreases as attention moves up the hierarchy closer to *the* self and away from the task. These findings are moderated by task and personal characteristics that are still poorly understood. As Kluger and DeNisi stated, there have been clear indications of feedback interventions producing negative effects since the beginning of the last century, but these have been largely ignored. In their opinion researchers and practitioners alike confuse their feelings that feedback is desirable with the question of whether feedback intervention benefits performance. The persistence of the positive view of feedback intervention is attributed to psychological, economic, and theoretical factors. Feedback is psychologically reassuring and people may like to obtain feedback [9].

However, the lack of a general theory is viewed as the major culprit. Without a comprehensive theory there is no way to integrate the vast and inconsistent empirical findings. We should not be surprised by the conclusion of Olde Bekkink et al. that no additional effect of explicit feedback could be demonstrated in the course examination subscores, whereas their initial study demonstrated higher scores following an interim assessment. Let us praise these results and use common sense. The outcome of the study by Olde Bekkink et al. saves busy professionals from using their time ineffectively while, as it turns out, students as self-directed learners seem to use feedback from various sources as they see fit. The authors called this *implicit* feedback. But we may also simply refer to it as: discussion after the interim assessment with peers, reading a book or a paper to find the correct answers, searching for information on internet databases, et cetera. The importance lies in students using various sources to improve their knowledge and apparently they do so.
